# Identifying candidate de novo genes expressed in the somatic female reproductive tract of *Drosophila melanogaster*

**DOI:** 10.1093/g3journal/jkad122

**Published:** 2023-06-01

**Authors:** Kaelina D Lombardo, Hayley K Sheehy, Julie M Cridland, David J Begun

**Affiliations:** Department of Evolution and Ecology, University of California Davis, Davis, CA 95616, USA; Department of Evolution and Ecology, University of California Davis, Davis, CA 95616, USA; Department of Evolution and Ecology, University of California Davis, Davis, CA 95616, USA; Department of Evolution and Ecology, University of California Davis, Davis, CA 95616, USA

**Keywords:** spermatheca, parovaria, seminal receptacle, evolution, *D. simulans*, *D. yakuba*

## Abstract

Most eukaryotic genes have been vertically transmitted to the present from distant ancestors. However, variable gene number across species indicates that gene gain and loss also occurs. While new genes typically originate as products of duplications and rearrangements of preexisting genes, putative de novo genes—genes born out of ancestrally nongenic sequence—have been identified. Previous studies of de novo genes in *Drosophila* have provided evidence that expression in male reproductive tissues is common. However, no studies have focused on female reproductive tissues. Here we begin addressing this gap in the literature by analyzing the transcriptomes of 3 female reproductive tract organs (spermatheca, seminal receptacle, and parovaria) in 3 species—our focal species, *Drosophila melanogaster*—and 2 closely related species, *Drosophila simulans* and *Drosophila yakuba*, with the goal of identifying putative *D. melanogaster*-specific de novo genes expressed in these tissues. We discovered several candidate genes, located in sequence annotated as intergenic. Consistent with the literature, these genes tend to be short, single exon, and lowly expressed. We also find evidence that some of these genes are expressed in other *D. melanogaster* tissues and both sexes. The relatively small number of intergenic candidate genes discovered here is similar to that observed in the accessory gland, but substantially fewer than that observed in the testis.

## Introduction

While the majority of new genes arise through various forms of gene duplication (Long et al. 2003), new genes may also arise from ancestrally nongenic DNA (Begun et al. 2006; Levine et al. 2006). Here we define these de novo genes as sequences producing transcripts that are located in ancestrally intergenic DNA and for which there is no evidence of transcription in outgroups. Such transcripts may be coding or noncoding. While putative de novo genes have been found in a variety of taxa, including *Drosophila* (Begun et al. 2006, 2007; Levine et al. 2006; Zhou, Zhang, et al. 2008), fish (Baalsrud et al. 2018; Zhuang and Cheng 2021), rodents (Heinen et al. 2009; Murphy and McLysaght 2012; Neme and Tautz 2013; Casola 2018), plants (Zhang et al. 2019; Jin et al. 2021), and fungi (Cai et al. 2008; Li et al. 2010; Carvunis et al. 2012; Vakirlis et al. 2018), our understanding of their possible evolutionary and functional importance remains rudimentary.

Early investigations of de novo genes in the *melanogaster* subgroup of *Drosophila* provided circumstantial evidence that they may often be expressed in male reproductive tract tissues (Begun et al. 2006, Levine et al. 2006). That conclusion was supported by subsequent work in *Drosophila melanogaster* (Zhou, Zhang, et al. 2008; Zhao et al. 2014; Heames et al. 2020). Orphan genes found in the *obscura* group of *Drosophila*, some of which may have de novo origin, were more likely to be retained if they were highly expressed and male-biased (Palmieri et al. 2014). Population level analysis of intergenic testis-expressed candidate de novo genes in *D. melanogaster* found a total of 142 segregating and 106 fixed genes (Zhao et al. 2014). While more conservative criteria reduced the number of candidates (Cridland et al. 2022), there were still over 100 polymorphic and 50 fixed genes. A recent investigation of intergenic and intronic candidate de novo genes expressed in the accessory gland + ejaculatory duct (a somatic male reproductive tissue) of *D. melanogaster* revealed 133 candidates, (84 intronic and 49 intergenic). Compared with intergenic testis-expressed de novo gene candidates from the same genotypes, intergenic AG-expressed genes tended to be fewer in number and expressed less consistently across genotypes (Cridland et al. 2022).

While the genetic and population level phenomena that might promote or facilitate expression of candidate de novo genes in male reproductive tract tissues remain obscure, the coevolutionary interactions between male and female reproduction raise interesting questions about whether the evolution of male-expressed genetic novelties is correlated with similar processes operating in female reproductive tract (FRT) transcriptomes. Here we begin addressing this question in an analysis of 3 somatic tissues of the *Drosophila* female reproductive tract, the parovaria, the seminal receptacle, and the spermatheca. These tissues are all crucial to female reproduction, including ovulation, fertilization, and sperm storage. The parovaria, or female accessory glands, have secretory functions required for fertilization and ovulation (Sun and Spradling 2012). The seminal receptacle is responsible for short term sperm storage (Fowler 1973); given the *D. melanogaster* mating system, the seminal receptacle may contain the sperm of multiple males (Manier et al. 2010). Spermathecae also serve as sperm storage organs, but are biased toward long-term storage specifically (Pitnick et al. 1999). The spermathecae also have secretory cells that participate in fertilization and ovulation pathways (Schnakenberg et al. 2011; Sun and Spradling 2013).

We chose these tissues because the potential for direct interaction of male products, including those produced by novel genes, with the female reproductive tract might generate selection favoring female-expressed genetic novelties. Because these female organs are small and poorly studied, and because de novo genes tend to be expressed at low levels, they are unlikely to have been discovered in previous work based on annotations or transcriptome analysis of whole animals or bulk female reproductive tracts. To begin to identify these potentially overlooked de novo genes, we characterized the transcriptomes of parovaria, spermatheca, and seminal receptacle from mated females from our focal species, *D. melanogaster,* and from 2 closely related species, *Drosophila simulans* and *Drosophila yakuba*. Using these data in combination with existing genomic resources, we identified putative *D. melanogaster* de novo genes expressed in these tissues and compared their attributes with those of candidate de novo genes previously identified from the study of male *D. melanogaster* reproductive tissues.

## Materials and methods

### Fly strains, data sets used, sequencing, and data processing

*Drosophila melanogaster* DGRP (*Drosophila* genetic reference panel) inbred strains from Raleigh, NC ((Mackay et al. 2012); RAL 304, 307, 360, 399, and 517) were raised at 25°C in a 12:12 light:dark cycle. In addition to these inbred lines, an F1 female genotype derived from crossing RAL 304 females × RAL 307 males was also used. Individual 3–5-day-old virgin females from lines RAL 304, RAL 307, RAL 360, RAL 399, or RAL 304/307 heterozygotes were placed in a vial with 2 RAL 517 males. Vials were observed, and the time of copulation recorded. Males were removed immediately after mating, with female reproductive tract tissues dissected within 3–5 h after the end of copulation, by which time the sperm storage organs are expected to be full (Fowler 1973). The dissected tissues were the parovaria, spermatheca, and seminal receptacle. Dissections were carried out 1 genotype at a time in a 9-well (3 × 3) depression plate. Wells were filled with ∼1-ml ice-cold phosphate-buffered saline (PBS). The initial dissection occurred in the middle well. First, the parovaria was removed from the rest of the reproductive tract and then transferred to a new well, where extra fat or other connecting tissue was removed; the parovaria were then moved to Trizol on ice. Forceps were checked for tissue contamination under the dissecting scope and then rinsed in ethanol. The spermathecae were then removed, rinsed in PBS, and transferred to Trizol on ice. Finally, the seminal receptacle was removed, rinsed in PBS, and then transferred to Trizol on ice. Dissections from ∼10 females were used for each organ and genotype. RNA was extracted from tissues using Trizol, and a cDNA library was produced using a SMART-Seq(R) v4 Ultra(R) Low Input RNA Kit for Sequencing (cat. 634896). Paired-end libraries were generated using the Nextera XT DNA Library Preparation Kit (FC-131-1024) and the Nextera XT Index Kit (FX-131-1001). These libraries were sequenced using 150 bp paired-end reads on an Illumina HiSeq4000 machine at the UC Davis Genome Center. The *D. simulans* and *D. yakuba* experiments were carried out as above, using lines Lara 10 (*D. simulans*, Sedghifar et al. 2016) and Tai18E2 (*D. yakuba*, Begun et al. 2007), respectively, with the exception being that females and males were derived from the same strain.

Trinity (Grabherr et al. 2011) was used to assemble transcripts from all reads, left and right, in FASTQ format, from each library. Reads from a total of 12 *D. melanogaster* libraries distributed across 5 genotypes and 3 tissues (not all genotypes were assayed for all 3 tissues) were pooled together and assembled (Supplementary Table 3). Default parameters were used. Raw reads were not trimmed prior to assembly, but instead, rigid downstream filtering (described below) was used to remove poor quality or repetitive sequence. This process aims to retain as much potentially useful data as possible. BLAST was also used to ensure that adapters had been properly removed from candidate de novo genes by searching for Illumina Nextera adapter sequences. Several candidate gene transcripts had adapter sequences appended to 1 end; these were manually trimmed. Trinity was also used at default parameters to assemble transcripts from RNA-seq reads from 8 tissue types (whole organism, reproductive tract, gonad, terminalia, thorax, abdomen, viscera, and head) from each of both sexes for 8 *Drosophila* species: *D. yakuba, D. ananassae, D. pseudoobscura, D. persimilis, D. willistoni, D. mojavensis, D. virilis,* and *D. grimshawi* (Yang et al. 2018). Additional details regarding these samples and reads can be accessed at the Gene Expression Omnibus (Edgar et al. 2002) under accession numbers GSE99574 and GSE80124. The resulting Trinity transcripts were used to create BLAST databases used to investigate the possibility that outgroup transcripts were homologous to *D. melanogaster* de novo gene candidates, thus falsifying the de novo gene hypothesis (see below).

### Criteria for identifying de novo gene candidates

While a small fraction of annotated *D. melanogaster* genes might have de novo origins, here we focus on currently unannotated candidate genes, as the properties of de novo genes—low expression levels, small size, and, by definition, absence from related species, makes them much less likely to be annotated. The procedure for identifying candidate de novo genes closely follows Cridland et al. (2022). Each of the Perl scripts utilized in the pipeline (Supplementary Fig. 1), as well as several intermediate steps that called external software or bash procedures, were called from a Python wrapper. Individual Perl scripts and Python wrapper are housed in a GitHub repository (https://github.com/kaelom/Dmel_DNG_Pipeline_2023).

All assembled transcripts were passed through a series of filters. First, we sought evidence for homology between our assembled transcripts and existing gene annotations or transcripts. To do this, we used BLAST (v. 2.10.1+, Altschul et al. 1990) to identify matches to existing gene annotations against reference genomes from *D. melanogaster* (v. 6.41)*, D. yakuba* (v. 1.05)*, D. simulans* (v. 2.02), and *D. ananassae* (v. 1.06) from the published Flybase reference (Gramates et al. 2022). Corresponding FASTA files contained records for 3′UTR, 5′UTR, intergenic, intronic, miRNA, miscRNA, ncRNA, pseudogene, transposon, tRNA, and CDS [Flybase; http://ftp.flybase.net/releases/FB2020_04/, all downloaded 2020 July 23, with the exception of *D. melanogaster*, http://ftp.flybase.net/releases/FB2021_04/dmel_r6.41/, downloaded 2021 August 16; (Thurmond et al. 2019)]. Databases created from these resources include the following: *D. melanogaster* chromosome and intergenic sequence, to ensure that transcripts are mapped to the reference sequence. To identify transcripts that correspond to existing genic annotations, we used BLAST against 3′-UTR, 5′-UTR, intronic, miRNA, miscRNA, ncRNA, pseudogene, transposon, tRNA, and CDS databases for every species available (see Supplementary Table 6 for the complete list). In all cases, a BLAST match was defined as 80% identity over at least 100 bp. Transcripts that matched any of these gene annotations in any of the 4 species were removed from further consideration.

To reduce the probability that the surviving *D. melanogaster* transcripts correspond to unannotated outgroup transcripts, we then compared them with a database of de novo assembled transcripts created from RNA-seq reads derived from 8 tissues (whole organism, gonad, reproductive tract, terminalia, thorax, viscera, head, and abdomen) from both sexes from each of the 8 aforementioned *Drosophila* species (Yang et al. 2018). All unannotated *D. melanogaster* FRT transcripts that returned a BLAST match, as defined above, to any of these resources, were removed.

The remaining *D. melanogaster* candidates were retained only if the transcript sequence was >300 bp long, the distance to the nearest exon annotation was >250 bp, and were intergenic (did not reside within annotated *D*. *melanogaster* introns). Additionally, candidates were required to be expressed at transcripts per million (TPM) ≥ 1 in at least 1 FRT library. TPMs were estimated using HISAT2 and StringTie [v2.2.1 (Kim et al. 2019); v2.1.4, (Pertea et al. 2015)]. First, HISAT2 was used to produce SAM files for each library by aligning our FRT raw reads with databases made from the *D. melanogaster* reference genome (v. 6.41), which were then converted to sorted BAM files with Samtools (v1.9, (H. Li et al. 2009). These BAM files, along with a *D. melanogaster* reference GTF (Flybase; http://ftp.flybase.net/releases/FB2020_04/dmel_r6.35/gtf/; downloaded 2021 August 16), updated to include Cridland et al. (2022) accessory gland candidates as well as our FRT de novo candidates, were used to create new GTF and abundance files for each library with StringTie, resulting in species and tissue expression estimates for each candidate transcript. For candidates with multiple isoforms, all isoforms were retained as long as all of these criteria were met for at least 1 isoform.

Finally, to provide further support for de novo origination and reduce the probability that incomplete and/or erroneous genome assemblies lead to errors in de novo gene identification, we performed a microsynteny analysis so that sequence homologous to the region corresponding to a de novo gene candidate could be identified in the orthologous regions of *D*. *simulans* and *D. yakuba*. To do so, we identified the annotated genes immediately upstream or downstream of each candidate. Then, the Flybase 2021 ortholog database (Flybase; http://ftp.flybase.net/releases/FB2021_02/precomputed_files/orthologs/; downloaded 2021 May 12) was used to identify the orthologs in the outgroups. A FASTA file containing those genes, the candidate, and 5 kb downstream and upstream of the region were then produced. BLAST analysis of these regions was then performed to identify these micro-syntenic regions in the reference chromosome databases for *D. simulans* and *D. yakuba.* A file was produced that contained the positions of orthologous matches, if they existed. Because of the small number of candidate genes identified here, we retain as weaker candidates those genes that failed this final synteny step with *D. simulans* or *D. yakuba*. Candidates that did not show positive evidence of syntenic regions in 1 or both outgroups were checked for upstream and downstream orthologs manually over larger genomic regions in the UCSC GenomeBrowser (Karolchik et al. 2003) to seek evidence of larger physical-scale candidate gene region orthology.

### Expression in existing transcriptome resources

To investigate evidence of candidate expression in other data sets, we focused on 3 resources: (1) a previously published transcriptome analysis of the female reproductive tract, (2) a community gene expression resource, and (3) our own collection of putative de novo genes expressed in the male accessory gland. First, we investigated expression of our candidate genes in RNA-seq data (McDonough-Goldstein et al. 2021) derived from 6 tissues from unmated and mated females: bursa, oviduct, seminal receptacle, spermathecae, parovaria, and the FRT-associated fat body. Tissues from mated females were collected 6 and 24 h post-mating. The *D. melanogaster* reference GTF (v6.41) was updated to include our de novo gene candidates. We then used StringTie (Pertea et al. 2015) with default parameters to estimate TPMs of the identified FRT candidate genes using the reads from McDonough-Goldstein et al. 2021. Second, we used reads from FlyAtlas2 (Leader et al. 2018; Krause et al. 2022), using the StringTie methods described above, to estimate expression of our candidate genes in a variety of *D. melanogaster* tissues. Finally, we used the same approach to investigate expression in our female data of previously identified candidate de novo genes expressed in the accessory gland + anterior ejaculatory duct of Raleigh inbred lines (Cridland et al. 2022).

### Coding potential

Coding potential of putative de novo genes was assessed with 2 different methods, coding potential calculator 2 (CPC2) and coding potential assessment tool (CPAT) (Wang et al. 2013; Kang et al. 2017). CPAT provides specific default parameters depending on the query species, therefore the default parameters for *Drosophila* were used. Settings for CPC2 are not dependent on the species being investigated. Browser versions of each tool were used at default parameters. CPAT and CPC2 each had their own proprietary coding potential cutoff of 0.39 and 0.5, respectively. CPAT's default minimum ORF length is 75 nucleotides, while CPC2 does not enforce a minimum.

Protein translations of these transcripts were run through SignalP 6.0, at default settings to investigate evidence of predicted signal sequences (Teufel et al. 2022), as strongly predicted signal sequences would provide support for the hypothesis that these transcripts are coding and have potential functions related to secretion.

## Results

### De novo genes identified and basic characteristics

We identified 61 candidate de novo transcripts (Supplementary File 1) associated with 35 de novo gene candidates (Table 1), which were expressed in *D*. *melanogaster* but for which we found no evidence of expression in *D. simulans* or *D. yakuba*. None of these candidates exhibited evidence of homology with transcripts observed from any tissue in any of the additional 7 *Drosophila* species examined (Methods). Of these 35 candidates, 32 had validated microsynteny with *D. simulans*, and 29 of those also had confirmed microsynteny with *D. yakuba*. This supports the proposition that generally the absence of mappable transcripts from *D. simulans and D. yakuba* cannot be explained by assembly gaps or errors, or by extremely high nucleotide divergence.

**Table 1.** Summarized candidate expression across data sets and tissues.

ID	Number of libraries expressed this report	Number of tissue × mating status expressed McDonough et al.	Number of tissues expressed FlyAtlas2	FRT tissue expression (This report, this report and McDonough et al., *McDonough et al*.)	FlyAtlas2 average TPMs			Max TPM this report
					Male	Female	3rd instar larvae	
TRINITY_DN18465_c0_g4	5	2	15	SR, ST, *Bursa*	Brain (7,11), crop (1.46), eye (4.80), head (2.72), TG (5.30)	Brain (7.00), crop (1.36), eye (4.41), head (2.95), ovary (1.46), TG (5.56), whole (1.49), carcass (1.02)	Larval CNS (9.86), larval trachea (1.56)	2.05
TRINITY_DN58663_c0_g1*^b^*	1	10	4	SR, *ST, Bursa, Oviduct*	Whole (1.40), MG (1.00), TG (1.10)	TG (1.40)	—	1.06
TRINITY_DN2173_c0_g1	3	3	2	ST	Eye (3.72)	Eye (1.07)	—	7.00
TRINITY_DN52147_c0_g1	4	3	—	SR	—	—	—	1.24
TRINITY_DN90_c0_g3	5	—	—	ST, PV	—	—	—	1.59
TRINITY_DN16805_c0_g2	2	3	—	ST	—	—	—	1.81
TRINITY_DN65427_c0_g1	1	—	3	ST	Brain (2.22)	Brain (1.66), TG (1.21)	—	1.09
TRINITY_DN40913_c0_g1	1	—	3	ST	Brain (1.22), TG (2.89)	TG (2.29)	—	1.23
TRINITY_DN7862_c0_g3	3	—	—	SR	—	—	—	3.29
TRINITY_DN5611_c0_g1	2	1	—	ST	—	—	—	2.47
TRINITY_DN4094_c0_g1*^b^*	2	1	—	SR	—	—	—	1.09
TRINITY_DN66768_c0_g1	1	—	2	ST	Anal Pad (6.27)	Anal Pad (5.38)	—	1.43
TRINITY_DN7830_c0_g1	1	—	2	ST	Anal Pad (3.93)	Anal Pad (8.41)	—	3.19
TRINITY_DN2143_c0_g2	1	—	2	ST	—	VST (1.81), MST (1.69)	—	1.63
TRINITY_DN3265_c1_g1	1	2	—	SR	—	—	—	1.26
TRINITY_DN72444_c0_g1	2	—	—	ST	—	—	—	1.68
TRINITY_DN7278_c0_g1	1	—	1	ST	AG (1.96)	—	—	1.07
TRINITY_DN24046_c0_g1	1	—	1	PV	—	Brain (1.03)	—	1.21
TRINITY_DN4410_c0_g1	2	—	—	SR, ST	—	—	—	1.22
TRINITY_DN4173_c1_g1	2	—	—	SR	—	—	—	1.50
TRINITY_DN11963_c0_g1	1	—	1	ST	Hindgut (1.18)	—	—	1.59
TRINITY_DN47998_c0_g1	2	—	—	SR	—	—	—	1.99
TRINITY_DN6533_c0_g1*^a^*	2	—	—	SR	—	—	—	1.81
TRINITY_DN22183_c0_g1	2	—	—	SR	—	—	—	1.65
TRINITY_DN41677_c0_g1	1	—	—	PV	—	—	—	1.01
TRINITY_DN15918_c0_g3	1	—	—	ST	—	—	—	1.27
TRINITY_DN22384_c0_g1	1	—	—	ST	—	—	—	1.45
TRINITY_DN13179_c1_g1	1	—	—	ST	—	—	—	1.40
TRINITY_DN43648_c0_g1*^a^*	1	—	—	SR	—	—	—	1.40
TRINITY_DN27150_c0_g4	1	—	—	SR	—	—	—	1.25
TRINITY_DN55991_c0_g1	1	—	—	SR	—	—	—	1.48
TRINITY_DN12518_c0_g2*^a^*	1	—	—	SR	—	—	—	1.03
TRINITY_DN65366_c0_g1*^b^*	1	—	—	SR	—	—	—	1.20
TRINITY_DN10735_c0_g1	1	—	—	SR	—	—	—	1.15
TRINITY_DN5533_c0_g1	1	—	—	ST	—	—	—	1.88

FlyAtlas2: numbers beside tissue label indicate the average TPM for all replicates of that tissue and sex. FRT Expression: it is important to note that for each candidate that was significantly expressed in a certain FRT tissue across our 12 libraries, that candidate was also significantly expressed in only the corresponding tissue(s) in the McDonough *et al.* FRT data set. FlyAtlas2 tissue abbreviation key: TG, thoracicoabdominal ganglion; MG, midgut, larval; CNS, larval central nervous system; VST, virgin spermatheca; MST, mated spermatheca; AG, accessory gland.

indicates microsynteny in *D. simulans* only.

indicates lack of microsynteny in both *D. simulans* and *D. yakuba*.

Most transcripts (44/61, 72%) had a simple structure, containing only 1 exon, though some contained up to 3. Similarly, most candidate genes were associated with a single isoform, though 14 had at least 2, and 1, TRINITY_DN4410, had as many as 7. Candidate transcript lengths, considering the longest isoform of each, range from 302 to 3477 bp, with an average length of 810 bp, similar to the mean length for previously published AG-expressed intergenic candidate genes (701 bp; Cridland et al. 2022) and testis-expressed candidate genes (935 bp; Zhao et al. 2014). These FRT-expressed candidate genes were distributed roughly homogeneously across chromosome arms (Supplementary Table 1).

### Quantification of expression of candidates in the FRT in DGRP strains

Per our filtering criteria, each candidate was expressed at a TPM ≥ 1 in at least 1 of the 12 tissue/genotype Raleigh inbred line combinations, though several (14/35, 40%) were expressed above this threshold in 2–5 libraries (Supplementary Table 1). Consistent with previous reports that de novo genes tend to be lowly expressed, the mean TPM of expressed candidate genes (including only observations of TPM ≥ 1) was only 1.57. Thus, many candidates were expressed at only slightly higher levels than the minimum TPM criterion for expression and only in 1 library. The maximum observed TPM was 7 (Table 1, Supplementary Table 1). All de novo gene candidates had nonzero TPMs in multiple libraries, 5 on average. To further summarize candidate de novo gene expression relative to expression levels of annotated genes, we calculated, per library, the number of annotated genes that were expressed over the maximum TPM observed among our candidates for that library. We found that, per library, 43% (in the RAL 307 spermatheca library) to 71% (in the RAL 304 parovaria library) of annotated genes exhibited TPMs greater than the maximum TPM of the most highly expressed candidate de novo gene observed in that library, providing further support for generally low expression of these candidate genes.

### Candidate expression in other female reproductive tract tissues

We took advantage of 2 existing RNA-seq resources to seek further evidence of expression of our 35 candidate genes. We used a published transcriptome analysis of the *D. melanogaster* female reproductive tract (McDonough-Goldstein et al. 2021), which included data from virgin and mated females (2 postcopulation time points, 6 h and 24 h) for the same 3 organs used here, as well as for the bursa, oviduct, and fat body.

In the female reproductive tract data from McDonough *et al*., we observed expression (mean TPM > 1 across biological replicates for each tissue/mating status) for 8 of 35 (23%) de novo gene candidates (Table 1). The maximum TPM of our candidates in any one of these libraries was 5.44. Most TPM ≥ 1 estimates were from either the spermatheca or seminal receptacle, and in many cases, these candidates also showed significant expression in those same tissues in our FRT data (Table 1). Often, expression in these tissues included numerous mating statuses (Supplementary Table 4). The correlated expression patterns between our data and those of McDonough *et al*. provide some additional support that these candidates are not simple technical artifacts.

### Expression of candidate de novo genes in FlyAtlas 2 data

To investigate evidence of broader candidate de novo gene expression in publicly available transcriptome data, we used the RNA-seq reads from FlyAtlas2 (Leader et al. 2018), which includes data from several adult male and female tissues, and 3rd instar larval tissues. Expression analysis was as described above, where TPM estimates for replicates and isoforms were pooled and averaged for each candidate. Genes were categorized as expressed in a tissue if mean TPM was ≥1. Eleven of 35 (31%) FRT-expressed candidates exhibited TPM ≥ 1 in at least 1 tissue in FlyAtlas2, with the maximum TPM from any 1 library being 12.63. Ten candidates were expressed in 1–4 of these tissues, but 1 candidate, TRINITY_DN18465_c0_g4, was expressed in 15 different tissues (Table 1, Supplementary Table 5).

### Expression of accessory gland expressed de novo genes in female reproductive tissues

Two of the organs used in our experiments, the spermatheca and parovaria, contain secretory cells (Allen and Spradling 2008; Mayhew and Merritt 2013). Because the male accessory gland is composed primarily of secretory cells (Wilson et al. 2017), we investigated whether previously identified accessory gland-expressed de novo gene candidates (Cridland et al. 2022) are expressed (TPM ≥ 1) in our FRT data. Twelve of the 133 AG-expressed candidates (∼9%) were also expressed in at least 1 FRT library (Supplementary Table 2). This supports the conclusion from the analysis of the McDonough *et al*. and FlyAtlas2 data that the FRT-expressed candidate de novo genes are not always tissue-specific, or even sex-specific in their expression. Whether or not the candidates expressed in FRT are biased toward secretory cell expression is an open question. Two of these 12 AG-expressed de novo genes that were also identified from our female tissues, TRINITY_DN4679_c0_g1 and TRINITY_DN42840_c0_g1, were also expressed (TPM ≥ 1) in the FRT data from McDonough-Goldstein et al. 2021.

Overall, of the 35 candidates pre-microsynteny validation, 24 (69%) were observed as expressed twice across our FRT data and that of McDonough *et al*. (Table 1). Of the 29 candidates genes with confirmed synteny to both outgroups, 12 (41%) were expressed at TPM ≥ 1 in more than 1 FRT library (Table 1), and 14 (48%) were expressed at mean TPM ≥ 1, in at least 1 of 2 previously published RNA-seq data sets (FlyAtlas 2 and McDonough-Goldstein et al. 2021; Table 1).

Given the heterogeneous genotypes, experimental conditions, and RNA-seq data production methods across these resources, this should be viewed as a lower bound on the repeatability of candidate gene transcript production in *D. melanogaster*.

### Coding potential

Two methods, CPAT and CPC2, were used to investigate coding potential of candidate transcripts. CPAT analysis revealed that while all 61 candidate transcripts had ORFs that exceeded the length minimum of 30 nucleotides, 2 transcripts, both isoforms of TRINITY_DN7862_c0_g3_(i1, i2) were identified as potentially coding. Isoform 1 of TRINITY_DN7862_c0_g3 was 428 bp long and single exon, while isoform 2 was 390 bp long and consisted of 2 exons. In our data, this gene was expressed only in the seminal receptacle of multiple lines: the RAL 304 (3.29 TPM), RAL 307 (2.94 TPM), and RAL 360 (1.18 TPM) (Supplementary Table 1).

CPC2 identified 4 candidate transcripts as potentially coding: 2 isoforms of TRINITY_DN16805_c0_g2_(i1, i3), TRINITY_DN47998_c0_g1_i1 and TRINITY_DN90_c0_g3_i1. TRINITY_DN16805 is a single exon candidate that has 3 isoforms overall. One of these isoforms is only 250 bp long; however, the other 2 isoforms, which were the 2 rated as potentially coding, are 1,105 and 1,147 bp long. This candidate is expressed at TPM ≥1 in the spermatheca of RAL 307 and RAL 307×RAL 304 F1 (1.81 TPM, 1.18 TPM). Both other potentially coding candidates are single isoform and single exon. TRINITY_DN47998 has a length of 617 bp and was expressed in 2 FRT libraries: RAL 307 × 304 F1 seminal receptacle (2 TPM) and RAL 360 seminal receptacle (1.37 TPM). TRINITY_DN90 is 486 bp long and was expressed at TPM ≥ 1 in 5 of our FRT libraries: RAL 304 spermatheca (1.14 TPM), RAL 304×RAL 307 F1 parovaria (1.40 TPM), RAL 304×RAL 307 F1 spermatheca (1.05 TPM), RAL 307 parovaria (1.58 TPM) and RAL 307 spermatheca (1.40 TPM) (Supplementary Table 1).

Two to 4 candidate transcripts (∼3–7%) had coding potential as indicated by 1 of the 2 programs. This is consistent with the low coding potential ratio observed in Cridland et al. (2022), where 163/165 (1%) of transcripts were called as potentially coding by CPAT. None of the ORFs associated with any candidates were predicted by SignalP to have a signal sequence, which is unsurprising considering the low number of potentially coding candidates.

## Discussion

Our investigation of putative de novo genes expressed in 3 organs of the *D. melanogaster* female reproductive tract, the parovaria, seminal receptacle, and spermatheca, revealed multiple similarities to candidate de novo genes expressed in the accessory gland + anterior ejaculatory duct (Cridland et al. 2022) of the same population. For example, the number of intergenic de novo gene candidates identified here (*n* = 35) is comparable with that for intergenic candidates observed in the accessory gland + anterior ejaculatory duct (*n* = 49) from a similar sample of the same population, though direct quantitative comparison is difficult due to differences between studies in the number of distinct organs sampled (2 in Cridland et al. (2022) and 3 here) and number of genotypes used (6 inbred genotypes in Cridland et al. 2022 vs 4 inbred genotypes and 1 heterozygous genotype here). Also similar to observations from the intergenic candidates expressed in the accessory gland + ejaculatory duct, none were expressed in all genotypes examined. Further similarities include their short length, simple organization, and low expression levels (Table 1, Supplementary Table 1), features shared more generally by candidate de novo genes in multiple taxa (reviewed in Van Oss and Carvunis 2019). While *D*. *melanogaster* testis-expressed candidate de novo genes share similarities with those expressed in the accessory gland and female reproductive tract, including their size, simplicity, and relatively low expression, they are much more abundant, and are expressed in a greater proportion of genotypes (Zhao et al. 2014, Cridland et al. 2022); 30% of testis candidates expressed in 1 genotype, compared with 60% of FRT candidates expressed in 1 genotype. While estimates of de novo gene number may be compromised by both false positives and false negatives, the similar material and approaches used across these tissues implies that observed similarities and differences between tissues are real. Whether the *Drosophila* female germline exhibits patterns of de novo gene expression similar to that of the male germline is an important unanswered question.

The expression of candidate genes in different female reproductive tract organs and in other tissues suggests that they are frequently not tissue- or organ-specific. Several are not sex-limited in expression, as in addition to expression in the parovaria, spermatheca, or seminal receptacle, where their transcripts are present in the male accessory gland and multiple tissues in both sexes. Whether this is a general property of *D. melanogaster*-specific de novo gene candidates is an open question. While computational analysis of the putative de novo genes identified here provides little support that most are protein-coding, firmer conclusions on this point await analysis of proteomic or ribo-profiling data (Zheng and Zhao 2022), or transgenic analysis of epitope-tagged individual candidates.

Finally, given their generally low expression levels and the fact they are not reliably expressed in all *D. melanogaster* genotypes, it remains to be seen whether genetic analysis will provide robust conclusions regarding the functions of these genes. Recent work on the transcriptional behavior of “naive” human DNA in yeast has suggested that eukaryotic DNA has inherent properties associated with the production of mature mRNAs (Luthra et al. 2022). Though the relevance of such observations to the attributes of endogenous intergenic DNA in *Drosophila* is unclear, this finding has potential relevance to the evolution of de novo genes. De novo genes, especially those that have low population frequencies, may be enriched for the products of some form of background (i.e. spurious) transcription (Begun et al. 2006), and if so, their presence and ability to be properly processed by the cell is not particularly informative about the probability that they have specific biological functions. Second, the properties of ancestrally intergenic DNA must necessarily lead to occasional production of spurious transcripts, a small subset of which may have or acquire functions that drive their spread through populations under directional selection (Begun et al. 2006, Zhao et al. 2014). Genetic and/or population genetic data (e.g. Zhao et al. 2014) will be necessary to elucidate the possible evolutionary and/or biological significance of these very young *Drosophila* genes.

## Supplementary Material

jkad122_Supplementary_Data

## Data Availability

Female reproductive tract sequences available at https://www.ncbi.nlm.nih.gov/sra under BioProject accession number PRJNA924827. Pipeline scripts and information can be found at https://github.com/kaelom/Dmel_DNG_Pipeline_2023. Supplementary File 1 contains the trimmed transcripts associated with the putative de novo gene candidates, as described above, in FASTA format. Supplemental material available at G3 online.

## References

[jkad122-B1] Allen AK , Spradling AC. The Sf1-related nuclear hormone receptor Hr39 regulates *Drosophila* female reproductive tract development and function. Development. 2008;135(2):311–321. doi:10.1242/dev.015156. 18077584

[jkad122-B2] Altschul SF , Gish W, Miller W, Myers EW, Lipman DJ. Basic local alignment search tool. J Mol Biol. 1990;215(3):403–410. 2231712 10.1016/S0022-2836(05)80360-2

[jkad122-B3] Baalsrud HT , Tørresen OK, Solbakken MH, Salzburger W, Hanel R, Jakobsen KS, Jentoft S. De novo gene evolution of antifreeze glycoproteins in codfishes revealed by whole genome sequence data. Mol Biol Evol. 2018;35(3):593–606. doi:10.1093/molbev/msx311. 29216381 PMC5850335

[jkad122-B4] Begun DJ , Lindfors HA, Kern AD, Jones CD. Evidence for de novo evolution of testis-expressed genes in the *Drosophila yakuba*/*Drosophila erecta clade*. Genetics. 2007;176(2):1131–1137. doi:10.1534/genetics.106.069245. 17435230 PMC1894579

[jkad122-B5] Begun DJ , Lindfors HA, Thompson ME, Holloway AK. Recently evolved genes identified from *Drosophila yakuba* and *D. erecta* accessory gland expressed sequence tags. Genetics. 2006;172(3):1675–1681. doi:10.1534/genetics.105.050336. 16361246 PMC1456303

[jkad122-B6] Cai J , Zhao R, Jiang H, Wang W. De novo origination of a new protein-coding gene in *Saccharomyces cerevisiae*. Genetics. 2008;179(1):487–496. doi:10.1534/genetics.107.084491. 18493065 PMC2390625

[jkad122-B7] Carvunis AR , Rolland T, Wapinski I, Calderwood MA, Yildirim MA, Simonis N, Charloteaux B, Hidalgo CA, Barbette J, Santhanam B, et al Proto-genes and de novo gene birth. Nature. 2012;487(7407):370–374. doi:10.1038/nature11184. 22722833 PMC3401362

[jkad122-B8] Casola C . From de novo to “de nono”: the majority of novel protein coding genes identified with phylostratigraphy are old genes or recent duplicates. Genome Biol Evol. 2018;10(11):2906–2918. doi:10.1093/gbe/evy231. 30346517 PMC6239577

[jkad122-B9] Cridland JM , Majane AC, Zhao L, Begun DJ. Population biology of accessory gland-expressed de novo genes in *Drosophila melanogaster*. Genetics. 2022;220(1):iyab207. doi:10.1093/genetics/iyab207. 34791207 PMC8733444

[jkad122-B10] Edgar R , Domrachev M, Lash AE. Gene expression omnibus: NCBI gene expression and hybridization array data repository. Nucleic Acids Res. 2002;30(1):207–210. doi:10.1093/nar/30.1.207. 11752295 PMC99122

[jkad122-B11] Fowler GL . Some aspects of the reproductive biology of *Drosophila*: sperm transfer, sperm storage, and sperm utilization. In: Caspari EW, editor. Advances in Genetics. New York: Academic Press; 1973. p. 293–360.

[jkad122-B12] Grabherr MG , Haas BJ, Yassour M, Levin JZ, Thompson DA, Amit I, Adiconis X, Fan L, Raychowdhury R, Zeng Q, et al Full-length transcriptome assembly from RNA-Seq data without a reference genome. Nat Biotechnol. 2011;29(7):644–652. doi:10.1038/nbt.1883. 21572440 PMC3571712

[jkad122-B13] Gramates LS , Agapite J, Attrill H, Calvi BR, Crosby MA, Dos Santos G, Goodman JL, Goutte-Gattat D, Jenkins VK, Kaufman T, et al Fly base: a guided tour of highlighted features. Genetics. 2022;220(4):iyac035. doi:10.1093/genetics/iyac035. 35266522 PMC8982030

[jkad122-B14] Heames B , Schmitz J, Bornberg-Bauer E. A continuum of evolving de novo genes drives protein-coding novelty in *Drosophila*. J Mol Evol. 2020;88(4):382–398. doi:10.1007/s00239-020-09939-z. 32253450 PMC7162840

[jkad122-B15] Heinen TJAJ , Staubach F, Häming D, Tautz D. Emergence of a new gene from an intergenic region. Curr Biol. 2009;19(18):1527–1531. doi:10.1016/j.cub.2009.07.049. 19733073

[jkad122-B16] Jin G , Ma P-F, Wu X, Gu L, Long M, Zhang C, Li D-Z. New genes interacted with recent whole-genome duplicates in the fast stem growth of bamboos. Mol Biol Evol. 2021;38(12):5752–5768. doi:10.1093/molbev/msab288. 34581782 PMC8662795

[jkad122-B17] Kang Y-J , Yang D-C, Kong L, Hou M, Meng Y-Q, Wei L, Gao G. CPC2: a fast and accurate coding potential calculator based on sequence intrinsic features. Nucleic Acids Res. 2017;45(W1):W12–W16. doi:10.1093/nar/gkx428. 28521017 PMC5793834

[jkad122-B18] Karolchik D , Baertsch R, Diekhans M, Furey TS, Hinrichs A, Lu YT, Roskin KM, Schwartz M, Sugnet CW, Thomas DJ, et al The UCSC genome browser database. Nucleic Acids Res. 2003;31(1):51–54. doi:10.1093/nar/gkg129. 12519945 PMC165576

[jkad122-B19] Kim D , Paggi JM, Park C, Bennett C, Salzberg SL. Graph-based genome alignment and genotyping with HISAT2 and HISAT-genotype. Nat Biotechnol. 2019;37(8):907–915. doi:10.1038/s41587-019-0201-4. 31375807 PMC7605509

[jkad122-B20] Krause SA , Overend G, Dow JAT, Leader DP. Flyatlas 2 in 2022: enhancements to the *Drosophila melanogaster* expression atlas. Nucleic Acids Res. 2022;50(D1):D1010–D1015. doi:10.1093/nar/gkab971. 34718735 PMC8728208

[jkad122-B21] Leader DP , Krause SA, Pandit A, Davies SA, Dow JAT. Flyatlas 2: a new version of the *Drosophila melanogaster* expression atlas with RNA-Seq, miRNA-Seq and sex-specific data. Nucleic Acids Res. 2018;46(D1):D809–D815. doi:10.1093/nar/gkx976. 29069479 PMC5753349

[jkad122-B22] Levine MT , Jones CD, Kern AD, Lindfors HA, Begun DJ. Novel genes derived from noncoding DNA in *Drosophila melanogaster* are frequently X-linked and exhibit testis-biased expression. Proc Natl Acad Sci U S A. 2006;103(26):9935–9939. doi:10.1073/pnas.0509809103. 16777968 PMC1502557

[jkad122-B23] Li D , Dong Y, Jiang Y, Jiang H, Cai J, Wang W. A de novo originated gene depresses budding yeast mating pathway and is repressed by the protein encoded by its antisense strand. Cell Res. 2010;20(4):408–420. doi:10.1038/cr.2010.31. 20195295

[jkad122-B24] Li H , Handsaker B, Wysoker A, Fennell T, Ruan J, Homer N, Marth G, Abecasis G, Durbin R. 1000 Genome Project Data Processing Subgroup. The sequence alignment/map format and SAMtools. Bioinformatics. 2009;25(16):2078–2079. doi:10.1093/bioinformatics/btp352. 19505943 PMC2723002

[jkad122-B25] Long M , Betrán E, Thornton K, Wang W. The origin of new genes: glimpses from the young and old. Nat Rev Genet. 2003;4(11):865–875. doi:10.1038/nrg1204. 14634634

[jkad122-B26] Luthra I , Chen XE, Jensen C, Rafi AM, Salaudeen AL, de Boer CG. Biochemical activity is the default DNA state in eukaryotes 2022. doi:10.1101/2022.12.16.520785. 38448573

[jkad122-B27] Mackay TFC , Richards S, Stone EA, Barbadilla A, Ayroles JF, Zhu D, Casillas S, Han Y, Magwire MM, Cridland JM, et al The *Drosophila melanogaster* genetic reference panel. Nature. 2012;482(7384):173–178. doi:10.1038/nature10811. 22318601 PMC3683990

[jkad122-B28] Manier MK , Belote JM, Berben KS, Novikov D, Stuart WT, Pitnick S. Resolving mechanisms of competitive fertilization success in *Drosophila melanogaster*. Science. 2010;328(5976):354–357. doi:10.1126/science.1187096. 20299550

[jkad122-B29] Mayhew ML , Merritt DJ. The morphogenesis of spermathecae and spermathecal glands in *Drosophila melanogaster*. Arthropod Struct Dev. 2013;42(5):385–393. doi:10.1016/j.asd.2013.07.002. 23872109

[jkad122-B30] McDonough-Goldstein CE , Borziak K, Pitnick S, Dorus S. *Drosophila* female reproductive tract gene expression reveals coordinated mating responses and rapidly evolving tissue-specific genes. G3 (Bethesda). 2021;11(3):jkab020. doi:10.1093/g3journal/jkab020. 33890615 PMC8063083

[jkad122-B31] Murphy DN , McLysaght A. De novo origin of protein-coding genes in murine rodents. PLoS One. 2012;7(11):e48650. doi:10.1371/journal.pone.0048650. 23185269 PMC3504067

[jkad122-B32] Neme R , Tautz D. Phylogenetic patterns of emergence of new genes support a model of frequent de novo evolution. BMC Genomics. 2013;14(1):117. doi:10.1186/1471-2164-14-117. 23433480 PMC3616865

[jkad122-B33] Palmieri N , Kosiol C, Schlötterer C. The life cycle of *Drosophila* orphan genes. Elife. 2014;3:e01311. doi:10.7554/elife.01311. PMC3927632 24554240

[jkad122-B34] Pertea M , Pertea GM, Antonescu CM, Chang T-C, Mendell JT, Salzberg SL. Stringtie enables improved reconstruction of a transcriptome from RNA-seq reads. Nat Biotechnol. 2015;33(3):290–295. doi:10.1038/nbt.3122. 25690850 PMC4643835

[jkad122-B35] Pitnick S , Marrow T, Spicer GS. Evolution of multiple kinds of female sperm-storage organs in *Drosophila*. Evolution. 1999;53(6):1804–1822. doi:10.1111/j.1558-5646.1999.tb04564.x. 28565462

[jkad122-B36] Schnakenberg SL , Matias WR, Siegal ML. Sperm-storage defects and live birth in *Drosophila* females lacking spermathecal secretory cells. PLoS Biol. 2011;9(11):e1001192. doi:10.1371/journal.pbio.1001192. 22087073 PMC3210755

[jkad122-B37] Sedghifar A , Saelao P, Begun DJ. Genomic patterns of geographic differentiation in *Drosophila simulans*. Genetics. 2016;202(3):1229–1240. doi:10.1534/genetics.115.185496. 26801179 PMC4788119

[jkad122-B38] Sun J , Spradling AC. Female reproductive glands play essential roles in reproduction that may have been conserved during evolution. Biol Reprod. 2012;87(Issue Suppl_1):347. doi:10.1093/biolreprod/87.s1.347.

[jkad122-B39] Sun J , Spradling AC. Ovulation in *Drosophila* is controlled by secretory cells of the female reproductive tract. Elife. 2013;2:e00415. doi:10.7554/eLife.00415. PMC3628084 23599892

[jkad122-B40] Teufel F , Armenteros JJA, Johansen AR, Gíslason MH, Pihl SI, Tsirigos KD, Winther O, Brunak S, von Heijne G, Nielsen H. Signalp 6.0 predicts all five types of signal peptides using protein language models. Nat Biotechnol. 2022;40(7):1023–1025. doi:10.1038/s41587-021-01156-3. 34980915 PMC9287161

[jkad122-B41] Thurmond J , Goodman JL, Strelets VB, Attrill H, Sian Gramates L, Marygold SJ, Matthews BB, Millburn G, Antonazzo G, Trovisco V, et al Flybase 2.0: the next generation. Nucleic Acids Res. 2019;47(D1):D759–D765. doi:10.1093/nar/gky1003. . 30364959 PMC6323960

[jkad122-B42] Vakirlis N , Hebert AS, Opulente DA, Achaz G, Hittinger CT, Fischer G, Coon JJ, Lafontaine I. A molecular portrait of de novo genes in yeasts. Mol Biol Evol. 2018;35(3):631–645. doi:10.1093/molbev/msx315. 29220506 PMC5850487

[jkad122-B43] Van Oss SB , Carvunis A-R. De novo gene birth. PLoS Genet. 2019;15(5):e1008160. doi:10.1371/journal.pgen.1008160. 31120894 PMC6542195

[jkad122-B44] Wang L , Park HJ, Dasari S, Wang S, Kocher J-P, Li W. CPAT: coding-potential assessment tool using an alignment-free logistic regression model. Nucleic Acids Res. 2013;41(6):e74. doi:10.1093/nar/gkt006. 23335781 PMC3616698

[jkad122-B45] Wilson C , Leiblich A, Goberdhan DCI, Hamdy F. The *Drosophila* accessory gland as a model for prostate cancer and other pathologies. Curr Top Dev Biol. 2017;121:339–375. doi:10.1016/bs.ctdb.2016.06.001. 28057306 PMC5224695

[jkad122-B46] Yang H , Jaime M, Polihronakis M, Kanegawa K, Markow T, Kaneshiro K, Oliver B. Re-annotation of eight genomes. Life Sci Alliance. 2018;1(6):e201800156. doi:10.26508/lsa.201800156. PMC6305970 30599046

[jkad122-B47] Zhang L , Ren Y, Yang T, Li G, Chen J, Gschwend AR, Yu Y, Hou G, Zi J, Zhou R, et al Rapid evolution of protein diversity by de novo origination in Oryza. Nat Ecol Evol. 2019;3(4):679–690. doi:10.1038/s41559-019-0822-5. 30858588

[jkad122-B48] Zhao L , Saelao P, Jones CD, Begun DJ. Origin and spread of de novo genes in *Drosophila melanogaster* populations. Science. 2014;343(6172):769–772. doi:10.1126/science.1248286. 24457212 PMC4391638

[jkad122-B49] Zheng EB , Zhao L. Protein evidence of unannotated ORFs in reveals diversity in the evolution and properties of young proteins. Elife. 2022;11:e78772. doi:10.7554/eLife.78772. PMC9560153 36178469

[jkad122-B50] Zhou Q , Zhang G, Zhang Y, Xu S, Zhao R, Zhan Z, Li X, Ding Y, Yang S, Wang W. On the origin of new genes in *Drosophila*. Genome Res. 2008;18(9):1446–1455. doi:10.1101/gr.076588.108. 18550802 PMC2527705

[jkad122-B51] Zhuang X , Cheng C-HC. Propagation of a de novo gene under natural selection: antifreeze glycoprotein genes and their evolutionary history in codfishes. Genes (Basel). 2021;12(11):1777. doi:10.3390/genes12111777. 34828383 PMC8622921

