# Supplementary figures and images for: Identifying candidate de novo genes expressed in the somatic female reproductive tract of Drosophila melanogaster
Source: G3 (Bethesda). 2023 Jun 1;13(8):jkad122. doi: 10.1093/g3journal/jkad122 (PMC10411569; doi:10.1093/g3journal/jkad122)

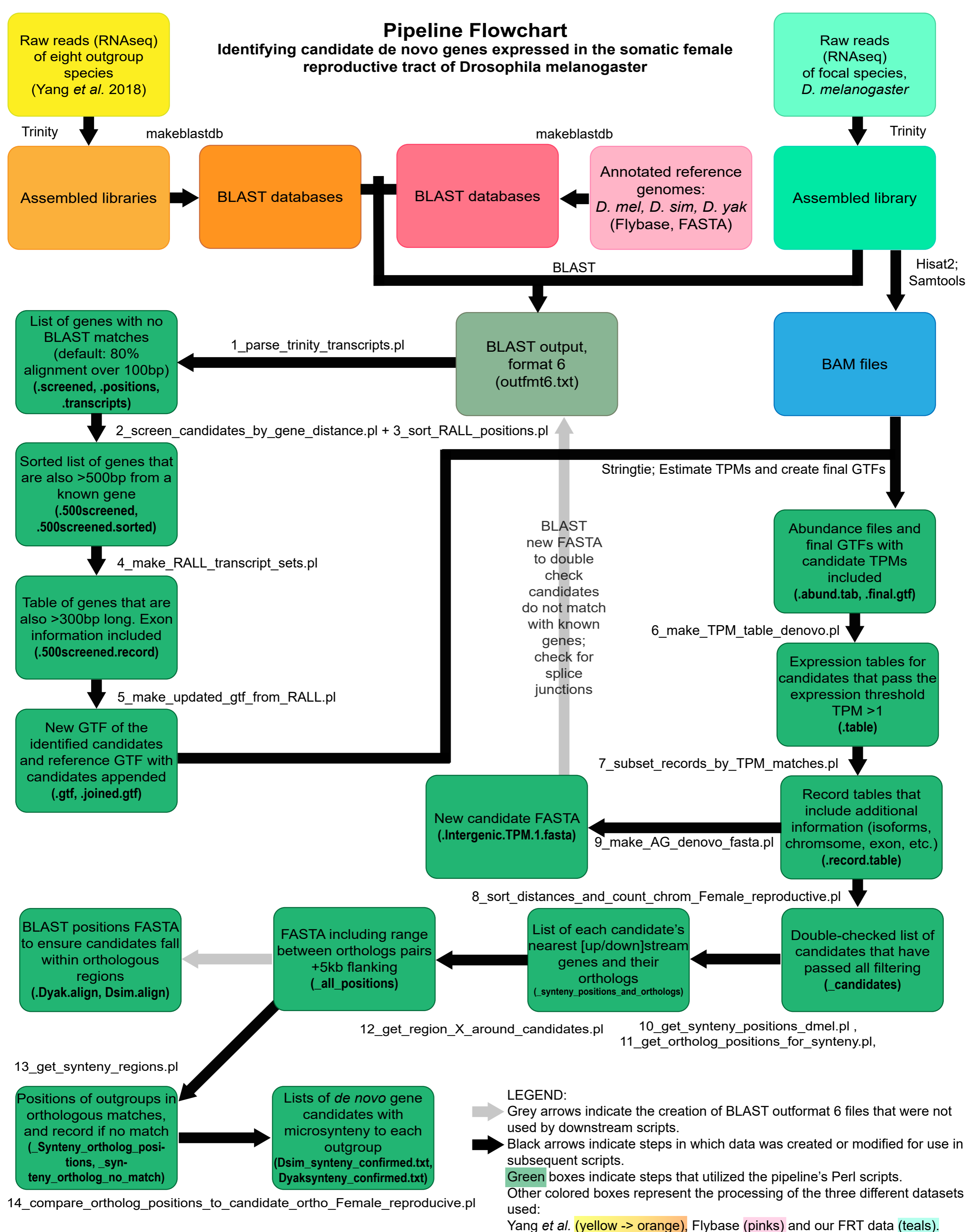

Supplement: jkad122_Supplementary_Data [file jkad122_supplementary_data.zip › Supplemental_Figure_1_G3-2023-404141.pdf]
